# Clinical Value of Fluorescent Lymphography with Indocyanine Green During Robotic Surgery for Gastric Cancer in Guided Lymph Node Dissection: A Systematic Review and Meta-Analysis

**DOI:** 10.3390/jpm16050243

**Published:** 2026-04-30

**Authors:** Dimitra V. Peristeri, Dimitrios N. Raptis, Ioannis Mantzoros, Dimitrios Schizas, Alexandros-Georgios I. Asimakopoulos, Eirini Papadopoulou, Georgios D. Lianos, Thomas Papaziogas, Vasileios Papaziogas

**Affiliations:** 1 Department of Bariatric and Upper GI Surgery, Musgrove Park Hospital, Somerset NHS Foundation Trust, Taunton, Somerset TA1 5DA, UK; 2 2nd Department of Surgery, General Hospital of Thessaloniki “G. Gennimatas’’, Aristotle University of Thessaloniki, 54635 Thessaloniki, Greece; draptis@hotmail.com (D.N.R.); papadopoulou.eirini242@gmail.com (E.P.); papaziog@auth.gr (V.P.); 3 4th Surgical Department, Papanikolaou General Hospital of Thessaloniki, Aristotle University of Thessaloniki, 54124 Thessaloniki, Greece; 4 1st Department of Surgery, National and Kapodistrian University of Athens, 17676 Athens, Greece; schizasad@gmail.com; 5 Department of Biostatistics, Hygiene and Epidemiology, School of Medicine, University of Ioannina, 45110 Ioannina, Greece; algioas@hotmail.com; 6 Department of Surgery, University Hospital of Ioannina, 45110 Ioannina, Greece; glianos@uoi.gr

**Keywords:** fluorescence, gastric cancer, robotic gastrectomy, indocyanine green, personalized surgical oncology

## Abstract

**Introduction**: Robotic gastrectomy is increasingly used in the surgical management of gastric cancer. Indocyanine green (ICG) near-infrared fluorescence imaging has emerged as a technique that enables real-time visualization of lymphatic drainage pathways, potentially facilitating more precise and individualized lymph node dissection. However, the clinical value of ICG-guided fluorescent lymphography during robotic gastrectomy remains incompletely established. **Methods**: A systematic review and meta-analysis were conducted in accordance with PRISMA guidelines. PubMed, Embase, Scopus, and the Cochrane Library were searched from database inception to 31 January 2026 for comparative studies evaluating ICG-guided fluorescent lymphography versus standard robotic gastrectomy for gastric cancer. Statistical analyses were performed using R (version 4.4.2) and the meta package. **Results**: Six studies, including 406 patients, met the inclusion criteria. Use of ICG was associated with a higher number of retrieved lymph nodes (mean difference [MD] 8.48; 95% CI 4.61–12.36; *p* = 0.001; I^2^ = 55.5%). Operative time was modestly shorter in the ICG group (MD −10.84 min; 95% CI −21.08 to −0.61; *p* = 0.038). There were no significant differences in intraoperative blood loss (MD −4.02 mL; *p* = 0.289), length of hospital stay (MD −0.82 days; *p* = 0.131), or postoperative complications (odds ratio 0.83; 95% CI 0.46–1.49; *p* = 0.534). **Conclusions**: ICG-guided fluorescence imaging during robotic gastrectomy is associated with increased lymph node retrieval and a small reduction in operative time without evidence of increased perioperative morbidity. Larger prospective studies are required to confirm these findings and to evaluate long-term oncologic outcomes.

## 1. Introduction

Gastric cancer (GC) constitutes a major global health issue, currently ranking as the fifth most prevalent malignancy and the fourth leading contributor to cancer-related deaths worldwide [1,2]. Most gastric cancers are adenocarcinomas, which are often diagnosed at an advanced stage due to the lack of symptoms in early disease. While advances in systemic and endoscopic therapies have expanded treatment options, surgery remains the mainstay of curative management, particularly in cases of locally advanced disease [3,4].

Radical gastrectomy combined with D2 lymphadenectomy (LND) remains the standard surgical approach for resectable GC, as it offers better locoregional control and improved long-term survival outcomes [3,4]. Current guidelines, including those from the American Joint Committee on Cancer (AJCC), recommend the retrieval of at least 16 lymph nodes (LNs), with higher counts—ideally 30 or more—associated with enhanced staging accuracy and prognostic stratification [5,6,7]. High-quality D2 LND has been shown to yield an average of 30 to 45 LNs, depending on the extent of gastrectomy, yet achieving this consistently remains technically demanding, particularly in patients with obesity or distorted anatomy [8,9,10,11].

Minimally invasive surgery (MIS), including laparoscopic and robotic approaches, has demonstrated favourable short-term outcomes and oncologic safety in the treatment of GC. However, adequate lymphadenectomy in MIS remains a challenge due to limitations in tactile feedback and visual contrast [12]. The application of indocyanine green (ICG)-enhanced fluorescence imaging using near-infrared (NIR) light has garnered interest as a viable method to circumvent existing limitations. ICG is a well-tolerated, FDA-approved dye that binds to plasma proteins and emits fluorescence under NIR light, enabling real-time visualisation of lymphatic pathways and vasculature. When injected endoscopically around the tumour prior to surgery, ICG can enhance intraoperative identification of LNs and facilitate a more precise dissection [13,14,15].

Although the optimal dosage, injection method, and timing vary across studies, the most commonly adopted protocol involves submucosal injection of 2.5 mg of ICG at four quadrants around the tumour, 24 h before surgery. ICG fluorescence imaging typically offers tissue penetration of approximately 0.5–1.0 cm and has demonstrated a favourable safety profile at commonly used doses [16,17,18]. Its adoption is increasing, particularly in robotic surgery, where platforms such as the da Vinci system offer integrated NIR imaging (“Firefly^®^” mode), allowing seamless transition between standard and fluorescence views [19].

Given these technical advantages, ICG-NIR imaging is increasingly used to enhance the quality of D2 LND during minimally invasive gastrectomy [20]. Early clinical studies—predominantly within laparoscopic cohorts—have suggested that fluorescence guidance may be associated with increased LN retrieval and enhanced intraoperative visualisation, although results remain heterogeneous and are largely derived from retrospective analyses [21]. Much of the existing evidence on ICG-guided lymphadenectomy originates from laparoscopic gastrectomy, with fewer studies specifically evaluating the robotic approach. In gastric cancer surgery, ICG fluorescence imaging may therefore contribute to a more personalized approach to lymphadenectomy by enabling surgeons to adapt dissection according to patient-specific lymphatic pathways and tumour localisation. The present study aims to assess the safety and effectiveness of ICG-guided fluorescence imaging in improving lymphadenectomy during robotic gastrectomy for gastric cancer.

## 2. Materials and Methods

A systematic search of Embase, PubMed/MEDLINE, Scopus, and the Cochrane Library was performed from database inception to 31 January 2026, following PRISMA guidelines (File S1) [22]. Google Scholar was not used as a primary database due to limited reproducibility; however, reference lists were screened to identify additional studies. The search strategy targeted three predefined concepts: (1) gastric cancer, (2) indocyanine green, and (3) robotic or minimally invasive gastrectomy. Because the topic is highly specialised, preliminary testing demonstrated that broader, sensitivity-oriented searches using exploded MeSH/Emtree terms, truncation, and proximity operators generated large volumes of non-relevant studies without identifying additional eligible records. Therefore, a specificity-oriented search strategy was intentionally chosen to optimise precision. This approach is acceptable for narrow surgical topics and was supplemented by manual screening of reference lists to minimise the risk of missed studies.

The aim was to identify studies (prospective or retrospective) and RCTs regarding the usage of Indocyanine Green during robotic gastrectomy for gastric cancer in guided lymph node dissection (D1 and D2 LND). The search strategy contained the search terms: (gastric cancer OR stomach cancer OR gastric OR cancers OR neoplasm) AND (indocyanine green OR green OR indocyanine OR wofaverdin, OR vophaverdin OR ujoveridin OR vofaverdin OR cardio-green OR cardio green) AND (robotic surgery OR robot-assisted gastrectomy OR minimally invasive gastrectomy OR robotic radical gastrectomy). In addition, the reference lists of all eligible studies were manually screened for further relevant publications identification. An English language limitation was applied. Risk of bias and methodological quality were assessed using the MINORS tool (Methodological Index for Non-Randomised Studies). This validated tool is specifically designed for evaluating non-randomised surgical intervention trials, using twelve criteria for comparative studies. The MINORS tool was used to assess the methodological quality of included non-randomised studies. A score of 18 or higher was used to designate studies as high-quality. The review protocol was specified a priori but was not prospectively registered in PROSPERO, which is acknowledged as a methodological limitation.

### 2.1. Study Selection Process

**Figure 1:**
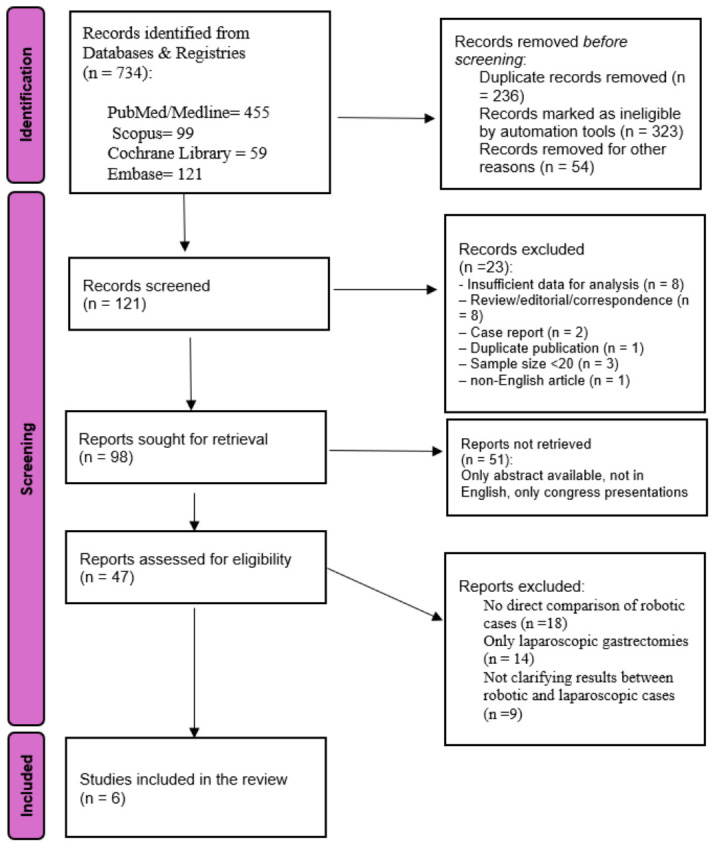
PRISMA flow diagram.

Prior to conducting the literature search, specific inclusion and exclusion criteria were defined. Following the PRISMA guidelines, the study selection process is illustrated in Figure 1. Eligible studies met the following conditions: (a) included patients diagnosed with resectable gastric cancer; (b) involved comparison between patients undergoing robotic radical gastrectomy (total or distal) with or without ICG tracer guidance; (c) provided complete datasets for analysis, avoiding duplication; and (d) encompassed studies such as retrospective, prospective or randomized controlled trials (RCTs). Studies excluded based on the following factors: (a) case reports, animal experiments, editorials, reviews, and correspondence; (b) insufficient or incomplete data for analysis; (c) duplicate publications by the same authors; (d) sample sizes smaller than 20 participants; (e) lack of direct comparison between robotic gastrectomy groups; and (f) articles not published in English.

### 2.2. Data Extraction

Data extraction involved collecting information such as study design and duration, year of publication, country where the study was conducted, the first author’s name, and patient demographics (including age, body mass index, sex, and ASA classification). Additional variables collected comprised preoperative clinical staging (T and N stage, including early versus locally advanced gastric cancer), histological findings from endoscopic biopsy, intraoperative parameters (such as surgical technique and duration of surgery and estimated blood loss), number of total lymph nodes retrieved, metastatic lymph nodes, and details of postoperative complications. The records were independently assessed by two reviewers (D.P. and D.R.)

### 2.3. Outcomes

The main objective was to evaluate the effect of ICG-NIR fluorescence imaging on the total lymph node yield in patients undergoing robotic radical gastrectomy. This parameter is widely used to determine the quality and oncological appropriateness of lymphadenectomy. Additional outcomes assessed included blood loss during surgery, to evaluate surgical safety and haemostatic control; total operating time, reflecting procedural efficiency; overall postoperative complications, defined as any adverse events occurring during the postoperative period and classified according to the Clavien-Dindo system when available [22]; and postoperative length of hospital stay (LOS), which provides an estimate of recovery trajectory and perioperative morbidity. Where necessary, median values were converted to means using validated statistical methods, and outcomes were standardised across studies to facilitate pooled analysis.

This study is a systematic review and meta-analysis of previously published data and did not involve direct contact with patients or access to individual patient-level records. In accordance with the policies of our institutions, formal Institutional Review Board (IRB) approval was not required for this type of study. No new human or animal research was conducted by the authors.

### 2.4. Statistical Analysis

Effect measures were expressed as odds ratios (ORs) for categorical outcomes and mean differences (MDs) for continuous data. For studies reporting continuous outcomes as mean and standard deviation (SD) or median and interquartile range (IQR), values were estimated using the following approximations: mean = median; SD = IQR/1.35. The pooled estimates and their 95% confidence intervals (CIs) were calculated using a DerSimonian–Laird random-effects model [23,24]. Heterogeneity among studies was assessed using the I^2^ statistic, with values of 25%, 50%, and 75% corresponding to low, moderate, and high heterogeneity, respectively, in addition to Cochran’s Q test [25]. Cochran’s Q test was used to assess statistical heterogeneity, with a significance level set at *p* < 0.10 to account for the low statistical power of the test when a small number of studies are included. Therefore, a *p*-value < 0.10 was considered indicative of statistically significant heterogeneity. Prediction intervals were calculated for all outcomes to demonstrate the expected effect range of a similar future study. Forest plots were used to visualise the effect estimates along with their 95% CIs across the included studies.

The small-study effect, as a proxy for publication bias, was planned to be assessed only if at least 10 studies were available per outcome, using visual inspection of funnel plots and Egger’s weighted regression test [26]. However, due to limited data, this analysis was not performed. All tests were two-tailed, and statistical significance was set at *p* < 0.05. A leave-one-out sensitivity analysis was performed to evaluate the influence of individual studies on the pooled weighted mean differences (WMDs) in the number of lymph nodes retrieved and to explore the potential impact of heterogeneity. This was performed by systematically excluding each study, one at a time, from the meta-analysis and recalculating the overall effect estimates. All analyses were conducted in R (version 4.4.2) using the meta package [27] and independently cross-checked for accuracy by a senior departmental statistician.

## 3. Results

### 3.1. Study Characteristics

The inclusion criteria were met by six studies and were included in the present meta-analysis [28,29,30,31,32,33]. The PRISMA flow diagram outlines the process of the study selection (Figure 1). All the key characteristics of the included studies are included in Table 1 and Table 2. Collectively, these studies encompassed 406 patients diagnosed with resectable gastric cancer, comprising 183 patients in the ICG group and 223 in the non-ICG group. Across all included studies, the ICG and non-ICG groups represented independent cohorts. Only 6 of the 406 patients included in the study received neoadjuvant chemotherapy (Table 2). In the study by Tian et al. [30], which included three arms (carbon nanoparticle suspension injection [CNSI], ICG, and conventional lymphadenectomy), only the ICG and conventional groups were included in the quantitative synthesis to allow a direct comparison between ICG-guided and non-ICG-guided robotic gastrectomy and to avoid unit-of-analysis errors. All patients in the study had similar characteristics. The CNSI group was excluded from the pooled analysis. Data on metastatic lymph node yield and nodal positivity, when reported, were extracted and summarised in Table S1.

Baseline equivalence between groups was reported or judged adequate through statistical tests in the majority of studies, supporting the validity of between-group comparisons. All studies were published between 2017 and 2023 and originated from Italy, Japan, China and Korea.

### 3.2. Details of ICG Administration

Details regarding ICG injection protocols are presented in Table 3. In 5 of the included studies, the ICG was administered via endoscopic submucosal injection, either intraoperatively [31] or within 24 h before surgery [28,29,30,32,33]. In all studies, the Da Vinci Si or Xi^®^ was the robotic platform used. The da Vinci Si and Xi systems were equipped with the Firefly^®^ system, which allows a simple finger click to switch between visible-light and NIR imaging without requiring equipment changes. The ICG injection concentration varied across the studies, as documented in Table 3; Importantly, no ICG-related adverse events were reported in any of the included studies.

### 3.3. Risk of Bias/Methodological Quality Assessment

The methodological quality of included studies was evaluated using the MINORS (Methodological Index for Non-Randomised Studies). A global score was calculated for each study by summing the individual item scores (ranging from 0 to 2 per item), with a maximum attainable score of 24 for comparative studies. Following re-evaluation of the original articles, MINORS scores for the items related to consecutive patient inclusion in the studies by Tian et al. [30] and Cianchi et al. [29]. were downgraded to reflect that consecutive recruitment was not clearly reported; however, most studies retained global MINORS scores ≥ 18, and the overall conclusions of the meta-analysis were unchanged. None of the included studies explicitly stated consecutive enrolment; therefore, the corresponding MINORS items were scored conservatively (Table S2). Notably, only one study reported an appropriate a priori power calculation.

### 3.4. Primary Outcome

**Figure 2:**
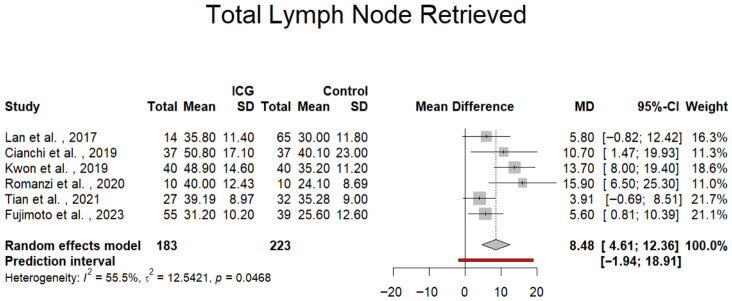
Forest plot showing the mean difference in the total number of lymph nodes retrieved between ICG and control patients. CI = Confidence Interval; ICG= Indocyanine Green; MD = Mean Difference [28,29,30,31,32,33].

For the primary outcome, a total of six studies involving 406 patients were included in the meta-analysis. The results indicate that patients undergoing robotic gastrectomy (total or distal) with ICG-NIR fluorescence guidance had a significantly higher mean number of LNs retrieved compared to those in the control group (mean difference [MD] = 8.48; 95% confidence interval [CI]: 4.61 to 12.36; *p* = 0.001). This suggests that ICG-enhanced imaging may improve the thoroughness of lymphadenectomy. However, the analysis revealed a moderate level of heterogeneity among the studies (*I*^2^ = 55.5%; *p* for Cochran’s Q = 0.0468), indicating some variability in effect estimates across studies. Moreover, the 95% prediction interval ranged from –1.94 to 18.91, which crosses zero, suggesting that the true effect in future studies may vary and could, in some cases, be nonsignificant (Figure 2). The statistically significant Q test (*p* = 0.0468) supports the presence of between-study heterogeneity beyond chance alone, consistent with the moderate I^2^ value observed.

### 3.5. Secondary Outcomes

#### Length of Stay (LOS)

**Figure 3:**
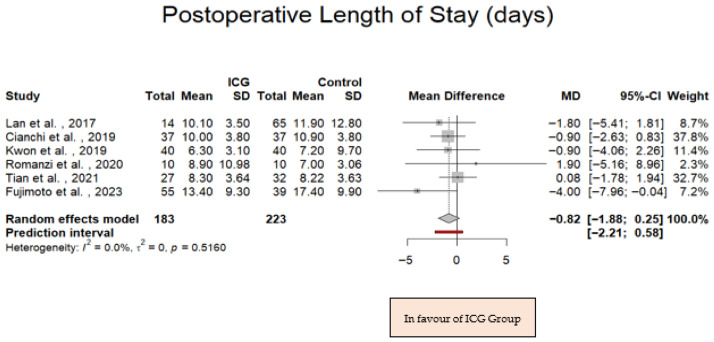
Forest plot presenting the mean difference in the postoperative length of stay among the ICG and control patients. CI = Confidence Interval; ICG= Indocyanine Green; MD = Mean Difference [28,29,30,31,32,33].

Analysis of combined data indicated comparable postoperative lengths of stay between the ICG-assisted and control groups. The mean difference (MD) was −0.82, 95% CI −1.88, 0.25; *p* = 0.131; *I*^2^ = 0%; *p* for Cochran Q = 0.516 (Figure 3), suggesting that the use of ICG did not contribute to prolonged hospital stay or delayed hospital discharge. The absence of heterogeneity (*I^2^* = 0%) indicates that this finding was consistent across all included studies.

### 3.6. Overall Postoperative Complications

**Figure 4:**
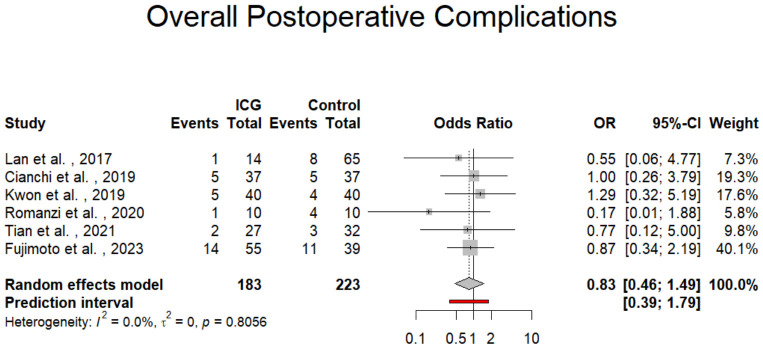
Forest plot presenting the odds ratio of the overall postoperative complications among the ICG and control patients. CI = Confidence Interval; ICG= Indocyanine Green; OR = Odds Ratio [28,29,30,31,32,33].

There was no statistically significant difference in the incidence of overall postoperative complications between the ICG and non-ICG groups. The pooled odds ratio (OR) was 0.83 (95% CI: 0.46 to 1.49; *p* = 0.534; *I*^2^ = 0%; *p* for Cochran Q = 0.8056; Figure 4), implying that the use of ICG fluorescence imaging did not increase or reduce the risk of complications following surgery. No heterogeneity further supports the reliability and uniformity of this result across studies.

#### 3.6.1. Perioperative Blood Loss

**Figure 5:**
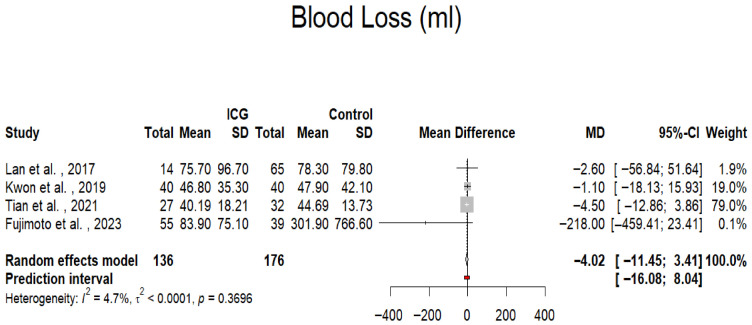
Forest plot presenting the mean difference in the blood loss among the ICG and control patients. CI = Confidence Interval; ICG = Indocyanine Green; MD = Mean Difference [30,31,32,33].

The effect of ICG on perioperative blood loss was also found to be statistically nonsignificant. Only four of the included studies reported outcomes on intraoperative blood loss [30,31,32,33]. The mean difference between groups was –4.02 mL (95% CI: –11.45 to 3.41; *p* = 0.289; *I*^2^ = 4.7%; *p* for Cochran Q = 0.3696; Figure 5), indicating minimal clinical impact. The low heterogeneity suggests that the amount of intraoperative bleeding was comparable between ICG-guided and conventional procedures across studies.

#### 3.6.2. Operative Time

**Figure 6:**
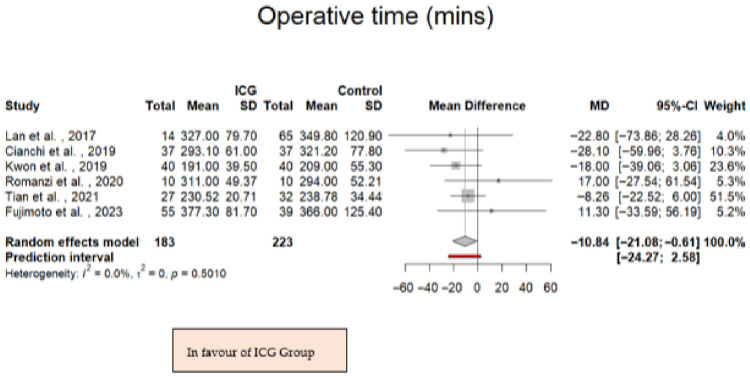
Forest plot presenting the mean difference in the operative time among the ICG and control patients. CI = Confidence Interval; ICG = Indocyanine Green; MD = Mean Difference [28,29,30,31,32,33].

A statistically significant difference in operative time was observed between the two groups. The pooled mean difference was –10.84 min with ICG (95% CI: –21.08 to −0.61; *p* = 0.038; *I*^2^ = 0%; *p* for Cochran Q = 0.501; Figure 6), suggesting that the use of ICG fluorescence imaging was associated with a modest reduction in operative time. The lack of heterogeneity further reinforces the consistency of operative times across the included studies.

### 3.7. Sensitivity Analysis

A leave-one-out sensitivity analysis was conducted to evaluate the influence of individual studies on the pooled WMDs in the number of lymph nodes retrieved and to explore the potential impact of heterogeneity. This was performed by systematically excluding each study, one at a time, from the meta-analysis and recalculating the overall effect estimates. The results of the leave-one-out sensitivity analysis demonstrated that the exclusion of any single study did not substantially alter the pooled WMDs or their corresponding 95% confidence intervals, indicating the robustness of the findings. Due to the limited number of included studies and the low observed heterogeneity, the influence of variability across studies on the primary outcome was considered statistically negligible.

## 4. Discussion

As minimally invasive surgery becomes increasingly refined, the application of ICG-enhanced NIR imaging in oncologic procedures has garnered significant attention. The present meta-analysis offers comprehensive and contemporary evidence regarding the role of ICG in enhancing LND during robotic gastrectomy for gastric cancer. Our findings demonstrate that ICG-guided surgery is associated with a significantly higher LN yield compared with conventional techniques. Given the established correlation between LN retrieval and accurate pathological staging, this may represent a clinically meaningful improvement, although long-term oncologic benefits cannot be determined from the available evidence. From the perspective of personalized medicine, fluorescence-guided lymphadenectomy represents an important step toward precision surgery. By enabling real-time visualization of patient-specific lymphatic drainage patterns, ICG imaging may allow surgeons to tailor lymph node dissection to the individual anatomy and tumour characteristics of each patient rather than relying exclusively on standardised anatomical templates.

Similarly, the pooled analysis demonstrated a directional trend towards shorter operative time with the use of ICG fluorescence imaging, although this finding should be interpreted cautiously given heterogeneity in study protocols and operative techniques. The observed effect may relate to more rapid intraoperative identification and confirmation of lymphatic anatomy during lymphadenectomy.

The increase in lymph node yield observed with ICG guidance is likely attributable to improved intraoperative visualization of lymphatic structures using near-infrared fluorescence. This advantage may be particularly relevant in patients living with obesity disease or increased visceral adiposity, where conventional visualisation is often limited [34]. By enhancing anatomical clarity and facilitating more efficient dissection, fluorescence guidance may contribute to both improved oncological quality and a reduction in operative duration. However, the magnitude of this effect is likely influenced by institutional experience, standardisation of ICG protocols, and team familiarity with fluorescence-guided techniques.

Significantly, ICG-guided surgery did not increase intraoperative blood loss, length of hospital stay, or postoperative complication rates. This supports its favorable safety profile and suggests that integration of fluorescence imaging into robotic workflows does not compromise perioperative outcomes, a critical factor for adoption in high-volume centers.

Robotic gastrectomy has gained increasing traction since its first clinical use in 2002. The da Vinci Surgical System offers technical advantages such as tremor filtration, three-dimensional magnified vision, and enhanced dexterity in confined spaces [35]. However, the lack of haptic feedback remains a notable limitation. ICG fluorescence imaging, particularly when integrated into robotic platforms via modes such as Firefly^®^, offers a visual surrogate for tactile cues by delineating lymphatic structures and vascular landmarks in real time [36]. This synergistic approach enhances precision, potentially reduces inadvertent tissue injury, and supports more complete lymphadenectomy. The emerging role of ICG fluorescence imaging in robotic D2 lymphadenectomy is of particular relevance given the technical complexity of this procedure [34]. While robotic platforms offer enhanced dexterity and high-definition visualisation, the absence of tactile feedback remains a key limitation. ICG near-infrared fluorescence imaging provides real-time visualisation of lymphatic pathways and vascular structures, thereby facilitating more precise and anatomically tailored lymph node dissection [35]. Emerging evidence suggests that fluorescence guidance may improve identification of lymph node stations, reduce the risk of incomplete dissection, and enhance oncological adequacy, particularly in anatomically challenging regions such as the suprapancreatic area. As robotic gastrectomy continues to evolve, integration of fluorescence imaging may contribute to standardisation of high-quality D2 lymphadenectomy and improve surgical reproducibility [35].

A recently published single-centre study by Fan et al. evaluated ICG-guided lymphadenectomy during robotic pylorus- and vagus nerve–preserving gastrectomy for early gastric cancer [36]. This study was not included in our quantitative synthesis because it was restricted to cT1N0 disease and primarily involved D1/D1+ lymphadenectomy within a function-preserving framework, which differs substantially from the radical robotic gastrectomy setting examined in the present analysis. In addition, the three-arm design incorporated a laparoscopic comparator, and the small ICG cohort did not demonstrate a statistically significant difference in total lymph node yield.

We also excluded the study by Jeong et al. [37] which assessed fluorescent lymphography during minimally invasive gastrectomy following chemotherapy. This investigation was not focused specifically on robotic surgery, as both robotic and laparoscopic cases were pooled without platform-specific stratification. Furthermore, it was a single-arm diagnostic performance study without a non-ICG robotic control group. Inclusion of these studies would have introduced substantial clinical and methodological heterogeneity and weakened the internal validity of our pooled robotic-specific estimates.

To our knowledge, this study constitutes one of the most up-to-date meta-analyses focusing exclusively on robotic gastrectomy and incorporating six comparative studies. Nevertheless, heterogeneity in ICG dose, timing of injection, and lymphadenectomy technique remains an important source of variability. Previous studies have highlighted the advantages of ICG fluorescence imaging in the context of laparoscopic gastric cancer surgery. In a comprehensive narrative review, Tan et al. analysed studies focusing predominantly on the use of ICG during laparoscopic gastrectomies [38]. Their findings suggested that ICG facilitates real-time visualisation of lymphatic drainage pathways and may enhance the thoroughness of lymph node dissection, thereby potentially improving surgical outcomes. However, the review did not include a formal meta-analysis, and the included studies demonstrated substantial heterogeneity in terms of control groups and study design. Furthermore, the review did not specifically focus on lymph node dissection as the primary outcome, but rather assessed the broader advantages of ICG technology, without a particular emphasis on robotic gastrectomies. A noteworthy systematic review by Deng et al. included 12 studies comprising a total of 1365 patients undergoing surgery for gastric cancer [39]. The analysis demonstrated that the use of ICG was associated with a significantly higher number of retrieved lymph nodes (weighted mean difference [WMD] = 7.67; 95% confidence interval [CI]: 4.73–10.62; *p* < 0.05), as well as a reduction in intraoperative blood loss (WMD = –10.28; 95% CI: –15.22 to –5.35; *p* < 0.05). However, the findings were mainly based on laparoscopic procedures rather than robotic-assisted surgery. Additionally, the presence of substantial heterogeneity and regional variation among the included studies may limit the generalizability and strength of the conclusions.

Another narrative review by Belia et al. suggested that NIR with ICG may assist surgeons in evaluating the adequacy of D1, D1+, and D2 lymphadenectomy by clearly delineating dissection boundaries and enhancing the visualisation of anatomical landmarks and fluorescent lymph node stations [40]. Their study stated that ICG technology holds particular promise in surgical education, as it may serve as a valuable tool for training less experienced surgeons in minimally invasive techniques, ensuring adequate lymph node retrieval and the performance of lymphadenectomies. However, their findings were neither systematic nor conclusive, and the review mainly included laparoscopic gastrectomies. Consequently, the results could not be directly applied to the context of robotic gastric cancer surgery. Similarly, a recent review by Guo et al. summarized the emerging role of ICG fluorescence imaging during robotic D2 lymphadenectomy [41]. However, that report was narrative and descriptive in nature, without a systematic search strategy, predefined eligibility criteria, formal risk-of-bias assessment, or quantitative meta-analysis. Heterogeneous study designs were discussed collectively, and no pooled robotic-specific comparative effect estimates were generated. Pang et al. also performed a meta-analysis encompassing 13 studies evaluating the role of ICG in LND during gastric cancer surgery [42]. The findings provided encouraging evidence that the use of ICG was significantly associated with a greater number of harvested LNs, without adversely affecting surgical safety in minimally invasive procedures. However, only four of the included studies specifically addressed robotic gastrectomy, and significant heterogeneity among these studies introduces a heightened risk of bias. Lastly, Zhang et al. conducted an extensive meta-analysis and literature review evaluating the clinical outcomes of ICG-guided lymphadenectomy in robotic gastric cancer surgery [35]. The analysis concluded that ICG-guided LND is a safe and effective approach, associated with an increased number of harvested lymph nodes and a reduction in operative time, without elevating the risk of intraoperative blood loss or postoperative complications. However, the study reported significant heterogeneity in the total number of lymph nodes dissected, largely attributable to the inherent limitations of the retrospective designs of the included studies. Moreover, due to the inclusion of a limited number of studies with relatively small patient cohorts, subgroup analyses could not be performed, thereby limiting the robustness and generalizability of the comparative findings.

The present analysis, to our knowledge, represents the most up-to-date meta-analysis focused exclusively on ICG-guided robotic gastrectomy, providing pooled quantitative estimates and methodological rigour to better define its clinical value. It builds upon this evidence base by focusing on robotic series and applying contemporary synthesis methods. Although the pooled effect estimate demonstrated an increase in lymph node retrieval, the 95% prediction interval crossed zero, indicating that the magnitude of benefit may vary across future clinical settings. This highlights the importance of local expertise, protocol standardization, and surgeon experience in achieving optimal outcomes with fluorescence-guided surgery.

Despite these encouraging findings, several limitations must be acknowledged. The overall number of included studies remains relatively small, and most were non-randomized, introducing potential selection bias. Variability in ICG dosage, timing of injection, and surgical approach may have contributed to heterogeneity. Furthermore, long-term oncologic outcomes such as disease-free and overall survival could not be assessed due to insufficient follow-up data.

Another limitation is the inconsistent reporting of nodal positivity and metastatic lymph node burden, which precluded quantitative synthesis of this clinically important outcome. Overall, the current evidence supports ICG-NIR fluorescence imaging as a safe adjunct to robotic gastrectomy that increases lymph node retrieval and a trend towards shorter operative time, which may vary according to institutional workflow and experience. Future prospective studies using standardised ICG protocols, with consistent reporting of both short-term surgical outcomes and long-term oncologic endpoints, are required to better define the clinical relevance of these findings. In addition, future studies may explore the role of ICG fluorescence imaging in assessing tissue perfusion and its potential impact on anastomotic integrity following gastrectomy.

## 5. Conclusions

In conclusion, ICG near-infrared fluorescence imaging appears to be a safe and useful adjunct to robotic gastrectomy and is consistently associated with increased lymph node retrieval. In addition, shorter operative times were observed in ICG-guided procedures, without an accompanying increase in intraoperative blood loss, length of hospital stay, or postoperative complication rates. These findings support a potential role for ICG in improving oncological quality and technical efficiency in lymphadenectomy. Nevertheless, heterogeneity across studies and nonsignificant prediction intervals suggest that the magnitude of benefit may vary between institutions. Further high-quality randomised controlled trials are needed to standardise ICG protocols, evaluate long-term oncologic outcomes, and confirm the generalisability of these findings. Until such data are available, ICG-guided imaging should be regarded as a promising adjunct in the surgical management of gastric cancer.

## Figures and Tables

**Table 1:** Characteristics of included studies.

Study	Year	Country	Study Interval	Study Design	Sample Size (I:C)	Operation Method	Imaging System
Romanzi [28]	2020	Italy	2017–2019	Single-centre, Prospective	10 vs. 10	RG	Da Vinci Si (Firefly^®^)
Cianchi [29]	2019	Italy	2014–2018	Single-centre, Prospective	37 vs. 37	RG	Da Vinci Si (Firefly^®^)
Tian [30]	2021	China	2019–2020	Single-centre, Retrospective	27 vs. 32	RDG	Da Vinci^®^ Xi System (Firefly^®^)
Lan [31]	2017	China	2011–2016	Single-centre, Retrospective	14 vs. 65	RG	Da Vinci Si (Firefly^®^)
Kwon [32]	2019	Korea	2012–2014	Single-centre, Prospective	40 vs. 40	RG	Da Vinci Si (Firefly^®^)
Fujimoto [33]	2023	Japan	2019–2022	single-centre, prospective, nonrandomised cohort	55 vs. 39	RDG	Da Vinci^®^ Xi or Si System (Firefly^®^)

RDG: Robotic Distal Gastrectomy, RG: Robotic Gastrectomy.

**Table 2:** Outcomes of Included Studies.

Study	Mean Operative Time Between Techniques (I:C) (min)	Type of LND	Number of LNs Retrieved	LOS (Days)	Blood Loss (mL) Median (IQR) (I:C)	Postoperative Complications (DC > III) (n)	Neo-Adjuvant Chemotherapy
Romanzi [28]	311 vs. 294	D2	40 vs. 24	8.9:7	N/A	3 vs. 2	2/20
Cianchi [29]	293 vs. 321	D2	50.8 vs. 40.1	10 vs. 10.9	N/A	5 vs. 5	4/74
Tian [30]	230.52 vs. 238	D2	39 vs. 35	8.3 vs. 8.8	40 vs. 44	2 vs. 3	None
Lan [31]	327 vs. 349.8	D1+ or D2	35.8 vs. 30	10.1 vs. 11.9	75.7 vs. 78.3	1 vs. 8	None
Kwon [32]	191 vs. 209	D1+ or D2	48.9 vs. 35	6.3 vs. 7.2	46.8 vs. 47.9	13 vs. 10	None
Fujimoto [33]	377.3 vs. 366	D2	31.2 vs. 25.6	13.4 vs. 17.4	83.9 vs. 301.9	14 vs. 11	None

**Table 3:** Characteristics of the ICG Injection.

Study	Way of ICG Injection	Time of Injection	ICG Injection Site	ICG Injection Concentration	ICG Injection Dose	Complications Related to ICG
Romanzi [28]	Endoscopy (submucosal)	18 h before surgery	4 points peritumoral, submucosal injection	1.25 mg/mL	2.4 mL in total	Nil
Cianchi [29]	Endoscopy (submucosa)	1 day before surgery	4 points peritumoral, submucosal injection	1.25 mg/mL	2 mL in total	Nil
Tian [30]	Endoscopy (submucosa)	1 day before surgery	4 points peritumoral, submucosal injection	N/A	2 mL in total	Nil
Lan [31]	Intraoperative subserosal injection OR Endoscopic	Intraoperative OR 1 day before surgery	4 points around the primary tumour, Chiba needle (18 gauge) for subserosal injection, endoscopic injection for submucosal injection.	2.5 mg/mL	2.4 mL in total	Nil
Kwon [32]	Endoscopy (submucosa)	22.4 h before surgery	4 points peritumoral, submucosal injection	1.25 mg/mL	2.4 mL in total	Nil
Fujimoto [33]	Endoscopy (submucosa)	1 day before surgery	4 points peritumoral, submucosal injection	1.25 mg/mL	2 mL in total	Nil

## Data Availability

No new data were created or analyzed in this study. Data sharing is not applicable to this article.

## Supplementary Materials

The following supporting information can be downloaded at https://www.mdpi.com/article/10.3390/jpm16050243/s1. Table S1. Number of Metastatic Lymph Nodes Retrieved in ICG versus non-ICG Groups, Table S2. Risk of Bias among included studies (MINORS rating score), File S1: PRISMA checklist [43].
