# Supplementary material for: Clinical Value of Fluorescent Lymphography with Indocyanine Green During Robotic Surgery for Gastric Cancer in Guided Lymph Node Dissection: A Systematic Review and Meta-Analysis
Source: J Pers Med. 2026 Apr 30;16(5):243. doi: 10.3390/jpm16050243 (PMC13208231; doi:10.3390/jpm16050243)
Supplement: Supplementary file 1 [file jpm-16-00243-s001.zip › jpm-4203622 Tables S1 and S2.pdf]

# Supplementary Material

**Supplementary Table S1: Number of Metastatic Lymph Nodes Retrieved in ICG versus non-ICG Groups.**

| *Study* | *Mean total lymph nodes retrieved per patient*<br><br>*ICG vs non-ICG (mean±SD)* | *Mean number of metastatic lymph nodes per patient*<br><br>*ICG vs non-ICG (mean±SD)* | *Total metastatic lymph nodes retrieved (proportion)*<br>*ICG vs non-ICG (%)* |
|---|---|---|---|
| **Romanzi[28]** | 40 vs 24 | N/A | N/A |
| **Cianchi[29]** | 50.8 ±17.1 **vs** 40.1±23.0, P<0.03 | 4.0±5.4 **vs** 4.4±6.8 | N/A |
| **Tian[30]** | 39.19 ± 8.97 vs 35.28 ± 9.00 | N/A | 49/1058 (4.63%) vs 67/1129 (5.93%) |
| **Lan[31]** | 35.8 vs 30 | N/A | N/A |
| **Kwon[32]** | 48.9 vs 35.2 | 5 vs 4 | 5/1956 (0.26%) vs 4/1408 (0.28%) |
| **Fujimoto[33]** | 31.2 vs 25.6 | N/A | N/A |

**Supplementary Table S2: Risk of Bias among included studies (MINORS rating score)**

| *Methodological item for non-randomized studies* | **Romanzi[28]** | **Cianchi[29]** | **Tian[30]** | **Lan[31]** | **Kwon[32]** | **Fujimoto[33]** |
|---|---|---|---|---|---|---|
| **A clearly stated aim** | 2 | 2 | 2 | 2 | 2 | 2 |
| **Inclusion of consecutive patients** | 2 | 1 | 0 | 1 | 2 | 2 |
| **Prospective collection of data** | 2 | 2 | 0 | 0 | 2 | 2 |

| | | | | | | |
|---|---|---|---|---|---|---|
| **Endpoints appropriate to the aim of the study** | 2 | 2 | 2 | 2 | 2 | 2 |
| **Unbiased assessment of the study endpoint** | 2 | 2 | 2 | 2 | 2 | 2 |
| **Follow-up period appropriate to the aim of the study** | 2 | 2 | 2 | 2 | 2 | 2 |
| **Loss to follow up less than 5%** | 2 | 2 | 2 | 2 | 2 | 2 |
| **Prospective calculation of the study size** | 0 | 0 | 0 | 0 | 0 | 2 |
| *Additional criteria in the case of comparative studies* | | | | | | |
| **An adequate control group** | 2 | 2 | 2 | 2 | 2 | 2 |
| **Contemporary groups** | 0 | 1 | 1 | 1 | 0 | 2 |
| **Baseline equivalence of groups** | 1 | 2 | 1 | 2 | 2 | 2 |
| **Adequate statistical analyses** | 2 | 2 | 2 | 2 | 2 | 2 |
| **SCORE** | **19** | **20** | **16** | **18** | **20** | **24** |

[The items are scored: 0 (not reported), 1 (reported but inadequate) or 2 (reported and adequate). The global ideal score is 16 for non-comparative studies and 24 for comparative studies.]
